# Ultrasound-guided fine needle aspiration of retropharyngeal lymph nodes after radiotherapy for nasopharyngeal carcinoma: a novel technique for accurate diagnosis

**DOI:** 10.1186/s40880-018-0286-z

**Published:** 2018-05-09

**Authors:** Long-Jun He, Chuanbo Xie, Yin Li, Lin-Na Luo, Ke Pan, Xiao-Yan Gao, Li-Zhi Liu, Jian-Ming Gao, Guang-Yu Luo, Hong-Bo Shan, Ming-Yuan Chen, Chong Zhao, Wei-Jun Fan, Ping Yang, Guo-Liang Xu, Jian-Jun Li

**Affiliations:** 1 State Key Laboratory of Oncology in South China, Collaborative Innovation Center for Cancer Medicine, Guangdong Key Laboratory of Nasopharyngeal Carcinoma Diagnosis and Therapy, Sun Yat-sen University Cancer Center, Guangzhou, 510060 Guangdong P. R. China; 2 Department of Endoscopy, Sun Yat-sen University Cancer Center, Guangzhou, 510060 Guangdong P. R. China; 3 Department of Cancer Prevention Research, Sun Yat-sen University Cancer Center, Guangzhou, 510060 Guangdong P. R. China; 4 Department of Melanoma Medical Oncology, The University of Texas MD Anderson Cancer Center, Houston, TX 77054 USA; 5 Department of Imaging and Invention Radiology, Sun Yat-sen University Cancer Center, Guangzhou, 510060 Guangdong P. R. China; 6 Department of Radiation Oncology, Sun Yat-sen University Cancer Center, Guangzhou, 510060 Guangdong P. R. China; 7 Department of Nasopharyngeal Oncology, Sun Yat-sen University Cancer Center, Guangzhou, 510060 Guangdong P. R. China; 8 Department of Pathology, Sun Yat-sen University Cancer Center, Guangzhou, 510060 Guangdong P. R. China

**Keywords:** Fine needle aspiration, Endoscopic ultrasonography, Retropharyngeal lymph node, Nasopharyngeal carcinoma

## Abstract

**Background:**

Enlarged retropharyngeal lymph nodes (RLNs) are very common in patients with nasopharyngeal carcinoma (NPC) undergoing radiotherapy. The most suitable treatment option for enlarged RLNs depends on the pathological results. However, RLN sampling is difficult and imminent in the clinic setting. We recently developed a novel minimally invasive technique termed endoscopic ultrasound-guided fine needle aspiration (EUS-FNA) for sampling RLN tissues sufficient for pathological or cytological diagnosis.

**Methods:**

We enrolled 30 post-radiotherapy patients with NPC with suspected RLN metastasis detected via magnetic resonance imaging (MRI). The EUS probe was introduced into the nasopharynx via the nostrils, and EUS was then used to scan the retropharyngeal space and locate the RLN in the anterior carotid sheath. EUS-FNA was subsequently performed. The safety and efficacy of using EUS-FNA to sample the RLN tissues were assessed.

**Results:**

Strips of tissue were successfully sampled from all patients using EUS-FNA. Of the 30 patients, 23 were confirmed to have cancer cells in the biopsied tissues via pathology or cytology examinations with 1 EUS-FNA biopsy session. The seven cases without confirmed cancer cells were subsequently reanalyzed by using another EUS-FNA biopsy session, and two more cases were confirmed possessing cancer cells. The other five patients without confirmed cancer cells were closely followed with MRI every month for 3 months. After follow-up for 3 months, three patients were still considered cancer-free due to the presence of RLNs with stable or shrinking diameters. The rest two patients who showed progressive disease underwent a third EUS-FNA biopsy procedure and were further confirmed to be cancer cell-positive. In the whole cohort reported here, the EUS-FNA procedure was not associated with any severe complications.

**Conclusion:**

EUS-FNA is a safe and effective diagnostic approach for sampling tissues from the RLNs in patients with suspected recurrent NPC.

**Electronic supplementary material:**

The online version of this article (10.1186/s40880-018-0286-z) contains supplementary material, which is available to authorized users.

## Background

Retropharyngeal lymph nodes (RLNs) are located in the retropharyngeal space, which is bordered anteriorly by the visceral fascia of the pharynx and posteriorly by the alar fascia. The retropharyngeal space is located lateral to the carotid sheath, which contains the internal carotid artery and internal jugular vein [1]. Enlarged RLNs are very common among patients with nasopharyngeal carcinoma (NPC) who undergo radiotherapy. It is vital to confirm whether the enlarged RLNs are a manifestation of NPC recurrence as it was related to the selection of  appropriate treatments. Close follow-up, including computed tomography (CT) [2], magnetic resonance imaging (MRI) [3–5], and positron emission tomography-CT [6, 7], can help to promptly detect an enlarged RLN. However, it is difficult to distinguish between benign lymph node hyperplasia and metastatic lymph nodes via imaging because of the high false-positive and false-negative rates. Biopsy is the gold standard technique with which to confirm RLN metastasis. However, RLN sampling is challenging because the RLNs are clinically impalpable and are generally located beyond the usual depth of neck dissection. In addition, RLNs are located adjacent to several crucial tissues and organs, such as the carotid sheath, nerves, and spine; hence, RLN sampling is associated with considerable surgical risk. Surgery via the mouth or through the neck is associated with increased trauma and a high risk of bleeding [7–10]. Most patients do not wish to undergo surgical resection as a diagnostic procedure. Therefore, it is vital to develop a simple and minimally invasive technique that can successfully obtain tissues or cells from an RLN to confirm the presence of RLN metastasis via pathology or cytology examinations, particularly when an enlarged RLN is isolated from patients with NPC who have previously undergone chemo-radiotherapy.

We recently developed a minimally invasive technique termed endoscopic ultrasound-guided fine needle aspiration (EUS-FNA) for sampling tissues from RLNs. When developing this procedure, we comprehensively considered the following advantages of EUS: (1) EUS is not only equipped with an ordinary optical scope to monitor mucosal lesions, but also has an ultrasound probe that can be placed into lumens or cavities (e.g., the nasopharynx, oropharynx, and hypopharynx) for the ultrasonographic examination of pharyngeal wall lesions and adjacent tissue conditions; (2) EUS allows for longitudinal sector scanning, which enables EUS-FNA of sample tissues from masses adjacent to the mediastinal wall, abdominal cavity, pancreas, and retroperitoneal spaces; and (3) endobronchial ultrasound-guided transbronchial needle aspiration (EBUS-TBNA) was recently developed for the assessment of tracheal/bronchial locations and hilar lymph nodes [11–13]. These EUS sampling techniques, including EUS-FNA [14, 15] and EBUS-TBNA, are considered to be safe and effective for obtaining tissues/cells for diagnosis. Hence, we proposed that an EUS probe can be introduced into the nasopharynx to facilitate ultrasound imaging, and that needle aspiration of the RLN (including parapharyngeal neoplasms) can also be performed.

In the present study, we evaluated the safety and efficacy of EUS-FNA sampling for the diagnosis of RLN metastasis in patients with NPC.

## Patients and methods

### Patients

We enrolled patients with NPC with suspected RLN metastasis after radiotherapy at Sun Yat-sen University Cancer Center, China, from January 2015 to June 2016. All patients met the following criteria: (1) presence of undifferentiated, non-keratinizing carcinoma upon initial diagnosis (World Health Organization, 1991 criteria [16]) and no evidence of metastasis in distant sites prior to radiotherapy; (2) administration of regular chemotherapy with cytotoxic agents, such as cisplatin, carboplatin, 5-fluorouracil, and paclitaxel, along with radiotherapy at standard doses (approximately 50–70 Gy) in the nasopharynx and neck that resulted in the lack of any local or distant lesions within 3 months after radiotherapy; (3) detection of an enlarged RLN via MRI during regular follow-up > 6 months after the end of radiotherapy and a minimum axial RLN diameter of > 5 mm; (4) no chemotherapy, radiotherapy, immunotherapy, or salvage surgery between the completion of radiotherapy and the MRI diagnosis of suspicious RLN metastasis; (5) no recurrent lesion in the nasopharynx on white light endoscopy and a pathological cancer cell-negative result on core biopsy; and (6) no other recurrent or metastatic lesions in local or distant organs.

The key raw data of this article have been uploaded to the Research Data Deposit public platform (https://www.researchdarta.org.cn), with the approval RDD number RDDA2017000153.

### Study protocol

**Fig. 1:**
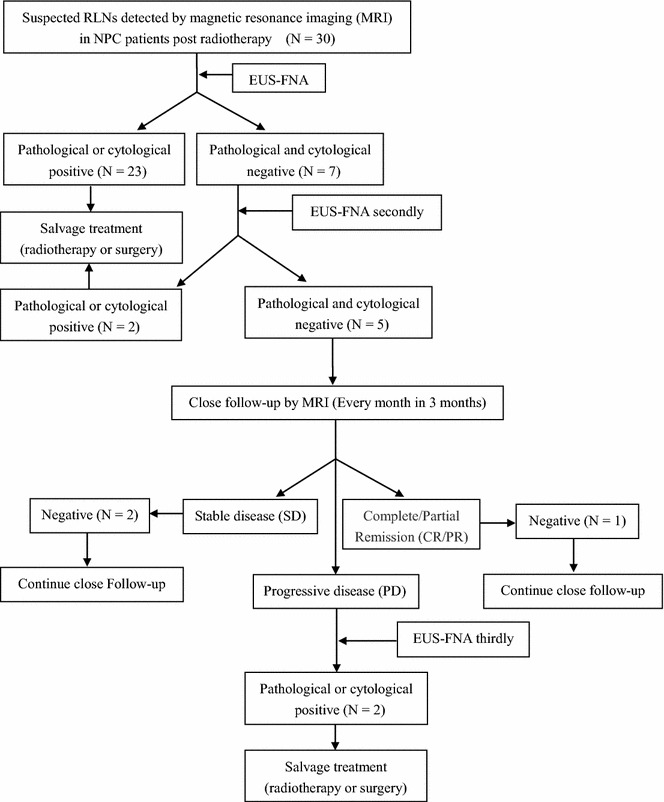
Study protocol for sampling of retropharyngeal lymph nodes (RLNs) with fine needle aspiration under endoscopic ultrasound guidance in post-radiotherapy patients with nasopharyngeal carcinoma. The RLNs were evaluated via magnetic resonance imaging using the New Response Evaluation Criteria in Solid Tumors: Revised RECIST guideline (version 1.1). European Journal of Cancer. 2009, (45): 228–247

The study protocol was approved by the institutional review board of Sun Yat-sen University Cancer Center, and informed consent was obtained from each patient. The EUS-FNA procedure was conducted in accordance with the approved guidelines. All included patients underwent EUS-FNA sampling from the RLNs. The sample tissues were then sent for pathologic or cytologic (ThinPrep cytologic test; Hologic, Marlborough, MA, USA) confirmation. The cancer cell-positive tissues were confirmed to be NPC metastasis in the RLN. The patients with cancer cell-negative tissues underwent a second EUS-FNA procedure to confirm the first EUS-FNA results. If the second EUS-FNA results were also negative, the patients were then closely followed up every month for 3 months by MRI. During the follow-up, if the patients showed stable or shrinking disease (partial response) in the RLN, they were still considered to be cancer cell-negative and underwent further follow-up [17, 18]. However, if the patients showed progressive disease in the RLN, a third EUS-FNA session was performed. Figure 1 presents the flow diagram of this study.

### EUS-FNA procedure

**Fig. 2:**
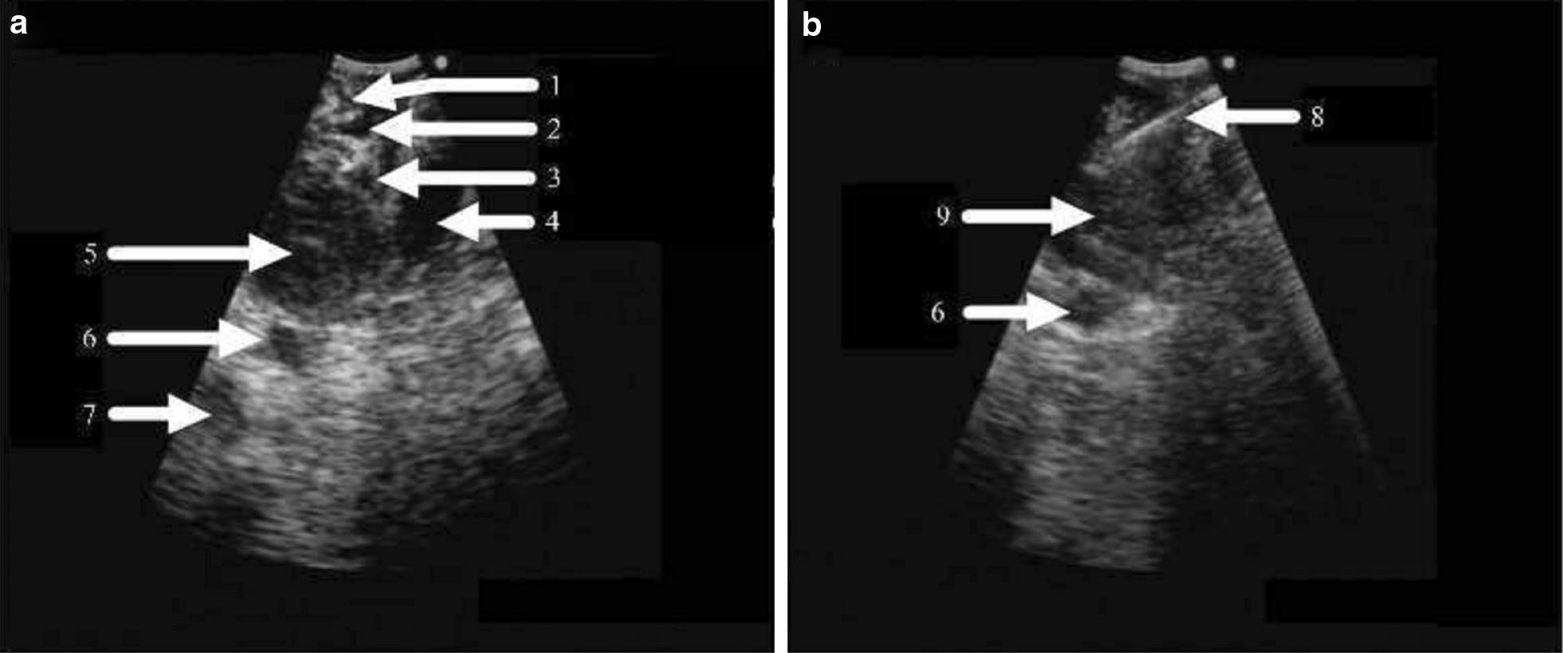
Endoscopic ultrasound (EUS) images of a retropharyngeal lymph node (RLN) in a patient with suspected recurrent nasopharyngeal carcinoma. **a** The enlarged RLN, which was roughly round and hypoechoic, was visualized by EUS. The RLN was located anterior to the carotid sheath, which included the internal carotid artery (ICA) and internal jugular vein (IJV); the RLN was also located beneath the lateral nasopharynx. **b** A fine needle was used to puncture the RLN for aspiration under real-time EUS guidance. 1 = tensor veli palatini; 2 = eustachian tube; 3 = levator veli palatini; 4 = longus capitis; 5 = RLN; 6 = ICA; 7 = IJV; 8 = needle; 9 = RLN

The schematic diagram and detailed description of our EUS-FNA procedure can be found in our previous case report [19]. In brief, an EUS probe (BF-UC 260F-OL8; Olympus Co., Tokyo, Japan) was introduced into the nasopharynx through the right nostril, and the nasopharynx and retropharyngeal space were scanned. A suspicious RLN metastasis was observed as a roughly round and homogeneously hypoechoic lesion in the retropharyngeal space that was located anterior to the carotid sheath, which contained the internal carotid artery and the internal jugular vein, and beneath the lateral nasopharynx, which contained the pharyngeal ostium of the eustachian tube, tensor veli palatini, and levator veli palatini (Fig. 2a). A dedicated 22-gauge needle (NA-201SX-4022; Olympus Co.) was then used to puncture the enlarged RLN under EUS guidance, and the needle was withdrawn under 20 ml of suction pressure (Fig. 2b). The puncture–suction action was repeated 30 times before the suction pressure was turned off, and the needle was removed from the patient. This entire EUS-FNA procedure was repeated at least three times until a strip of tissue was obtained. A video of the EUS-FNA procedure has been provided as Additional file 1: Video S1 (https://youtu.be/_3tcjv9GI9o). The obtained tissue samples were sent for pathologic examination, and the supernatant fluid was used for the ThinPrep cytologic test.

### Safety and efficacy assessment

Safety was evaluated according to whether EUS-FNA-related bleeding, subcutaneous emphysema, choking, dyspnea, abnormal sensation, extremity paralysis, or hemiplegia occurred during or after the EUS-FNA procedure. Efficacy was evaluated according to whether tissues or cells were successfully obtained from the RLNs using EUS-FNA and whether the tissue or cell samples were adequate for pathological or cytological tests.

## Results

### Characteristics of the included patients

In total, 30 patients with NPC with suspected recurrent RLN metastasis after radiotherapy were enrolled. The mean age of the patients was 47.2 years (standard deviation, 11.2 years). Most of the patients were male (88.3%), had lymph node metastasis (90%), and had undergone two-dimensional radiotherapy (60.0%). Other sociodemographic and clinical characteristics of the 30 patients with NPC are shown in Table 1.

**Table 1:** Characteristics of 30 patients with nasopharyngeal carcinoma with suspicious RLN metastasis

Characteristics	n (%)
Sex	
Male	25 (83.3)
Female	5 (16.7)
T stage of initial diagnosis on MRI	
T1	
T2	12 (40.0)
T3	13 (43.3)
T4	5 (16.7)
N stage of initial diagnosis on MRI	
N0	3 (10.0)
N1	14 (46.7)
N2	8 (26.7)
N3	5 (16.7)
RLN metastasis detected in the initial diagnosis on MRI	
Yes	19 (63.3)
No	11 (36.7)
Recurrent disease site detected via MRI	
Right lateral RLN	13 (43.3)
Left lateral RLN	16 (53.3)
Medial RLN	1 (3.3)
Previous chemo/radiotherapy regime	
Neoadjuvant chemotherapy plus radiotherapy	7 (23.3)
Radiotherapy plus adjuvant chemotherapy	6 (20.0)
Concomitant chemo-radiotherapy	16 (53.3)
Radiotherapy only	1 (3.3)
Previous chemotherapy agent	
Cisplatin only	16 (53.3)
5-flurouracil + cisplatin	8 (26.7)
Paclitaxel + carboplatin	5 (16.7)
Previous radiotherapy	
Two-dimensional radiotherapy	18 (60.0)
Three-dimensional conformal radiotherapy	6 (20.0)
Intensity-modulated radiotherapy	6 (20.0)
Duration between chemo-radiotherapy and detection of suspicious recurrent RLN	
6–12 months	6 (20.0)
1–3 years	13 (43.3)
4–5 years	5 (16.7)
5–10 years	5 (16.7)
> 10 years	1 (3.3)

*RLN* retropharyngeal lymph node, *MRI* magnetic resonance imaging

### Safety of EUS-FNA

The EUS-FNA procedure was smoothly conducted in all enrolled patients. No severe complications, such as bleeding, subcutaneous emphysema, choking, dyspnea, abnormal sensation, extremity paralysis, or hemiplegia, were noted. Furthermore, no obvious changes in vital signs were observed during or after EUS-FNA.

### Efficacy of EUS-FNA in acquiring RLN tissue

**Fig. 3:**
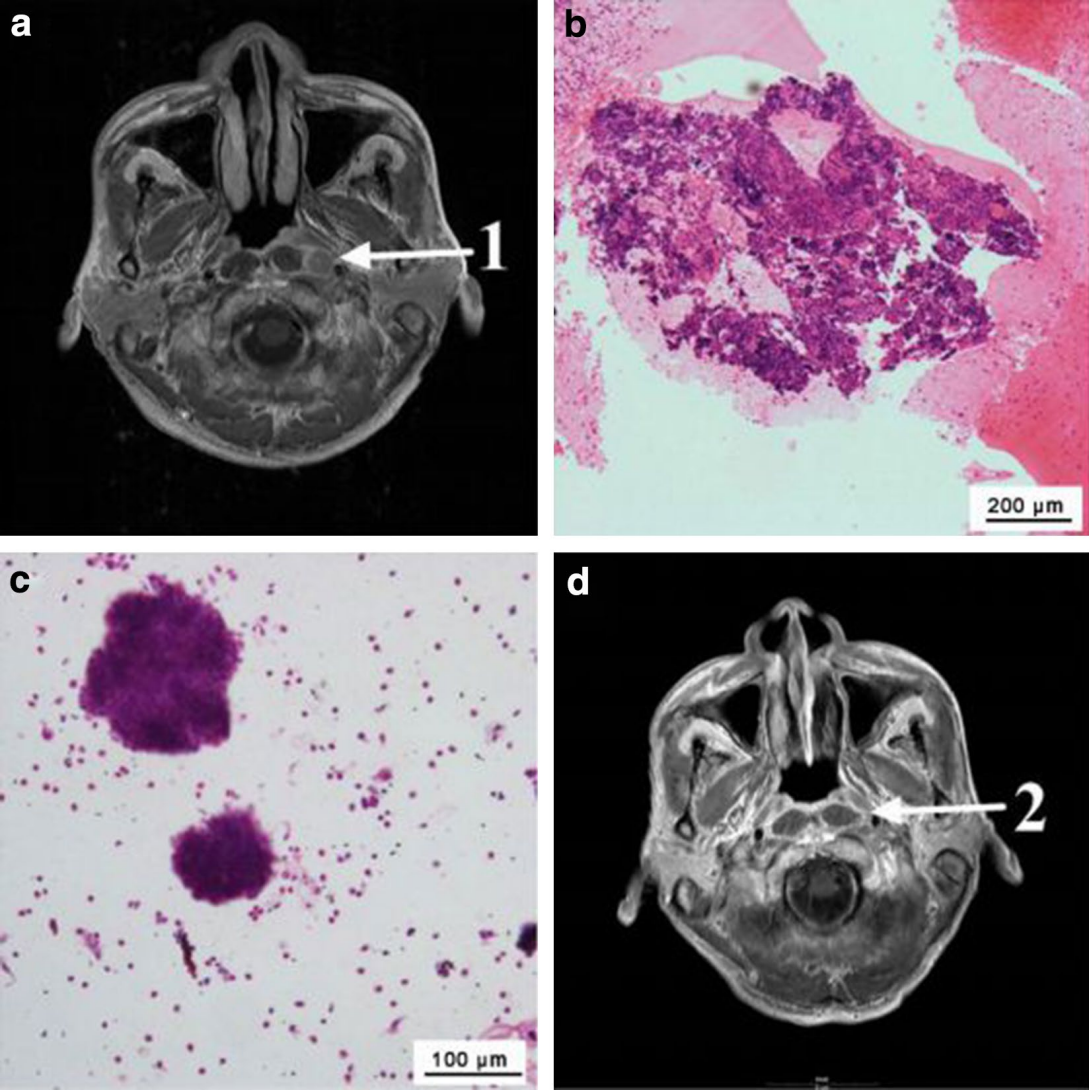
Representative case of a cancer-positive result in the retropharyngeal lymph node (RLN) confirmed during the first endoscopic ultrasound-guided fine needle aspiration (EUS-FNA) sampling session (Patient #11). **a** T1-weighed contrast-enhanced magnetic resonance imaging (MRI) revealed an enlarged left RLN in a patient with nasopharyngeal carcinoma. **b** Pathologic examination indicated cancer-positive results after a single EUS-FNA sampling session. On biopsy, the tumor showed sheets of cells with a characteristic syncytial pattern of growth, large nuclei, coarse chromatin, and conspicuous nucleoli. **c** Cytologic examination results indicated cancer-positive results after a single EUS-FNA sampling session. Cytological smears showed cohesive clusters of uniform large tumor cells associated with lymphocytic infiltration. Round or oval vesicular nuclei with a syncytial appearance, conspicuous nucleoli, and finely granular cytoplasm were noted. **d** The patient exhibited a complete response after salvage radiotherapy. No tumor was noted on MRI. 1 = pre-treatment RLN; 2 = post-treatment RLN

The RLN could be easily visualized through monitoring via real-time EUS. Recurrent RLN metastasis generally appeared as a round and homogeneously hypoechoic lesion in the retropharyngeal space, located anterior to the carotid sheath (Fig. 2a, b). The tissues or cells from the RLN of all patients were sampled successfully using EUS-FNA. The pathologic or cytologic results indicated squamous cell recurrence in the RLN in 23 patients following a single EUS-FNA session. Figure 3 presents the MRI findings from one such patient **(**Patient #11). MRI showed that the patient had an enlarged RLN (Fig. 3a). After sampling with EUS-FNA, both the pathologic and cytological examination results indicated cancer cell positivity (Fig. 3b, c). Thus, the patient was confirmed to have RLN metastasis. After salvage radiotherapy, the patient exhibited a complete response on MRI (Fig. 3d). The other seven patients without confirmed cancer cells underwent another EUS-FNA biopsy session for repeated sampling. Two cases were found to be cancer cell-positive and were diagnosed as RLN metastasis.

After two EUS-FNA sampling sessions, the other five patients without confirmed cancer cells were closely followed and re-examined with MRI every month. During the 3 months of follow-up, three patients were still considered to be cancer-free because of stable (two patients) or reduced (one patient) diameters of the RLNs. The rest two patients who exhibited progressive disease (continued enlargement of the RLN on MRI during the follow-up) were suspected to have metastasis. EUS-FNA biopsy sampling from the RLN was conducted again for these two patients, and pathological examination further confirmed both patients having RLN metastasis.

## Discussion

Based on our previous successful experience with EUS-FNA, we herein examined 30 patients with suspected RNL metastasis after radiotherapy to further evaluate the safety and efficacy of EUS-FNA sampling from RLNs for the diagnosis of metastasis. First, we found that no adverse events occurred during or after EUS-FNA in any of the patients, which suggests that EUS-FNA sampling from the RLNs was safe. Second, in our study, the RLN tissues or cells could be successfully obtained via EUS-FNA from all patients, and most recurrent cases could be confirmed in only one EUS-FNA session; this suggests that this method is effective for sampling and can confirm RLN metastasis in patients with NPC. Moreover, the quick diagnosis helped these patients to receive timely corresponding treatment. Most importantly, if the patients who were considered to be negative for cancer cells after two EUS-FNA sessions exhibited progressive disease during follow-up, then EUS-FNA sampling could be performed again for diagnosis (i.e., 2 of the 30 included patients underwent EUS-FNA twice). Thus, our study shows that EUS-FNA is a very reliable method for sampling tissue from the RLNs and for diagnosing patients with suspected RLN metastasis.

Surgical resection and CT-guided needle biopsy are two reported methods for sampling tissue from the RLNs. However, surgical resection is rarely accepted by patients as a diagnostic procedure because of the associated major trauma, high risk of bleeding, and long-term sequelae. Su et al. [20] assessed the use of CT-guided needles for sampling RLN tissue. The disadvantages of this approach were apparent: the procedure was difficult to manipulate and time-consuming; the needle path via the mandibular region was relatively long; the needle bypassed some vital structures such as the carotid sheath, which increased the surgical risk; and CT guidance did not enable real-time guidance and required frequent adjustment of the needle direction and depth, which increased the risk of severe adverse events. Therefore, few reports of CT-guided biopsies of RLN tissue or retropharyngeal neoplasms via the mandible have been reported to date. Compared with surgical resection and CT-guided biopsies, our novel EUS-FNA technique has a shorter puncture path, is easier to manipulate, is less time-consuming, and has a high success rate for RLN tissue sampling. Safety is another advantage of our new technique. Because aspiration is performed via the nasopharynx, the aspiration needle avoids passing adjacent to organs such as the carotid sheath and cranial nerves during EUS-FNA. Hence, our new technique might be of great clinical use in diagnosing RLN metastasis.

However, we also realize that our EUS-FNA sampling technique for the RLNs has some limitations. No technical ultrasonic endoscope has been developed for the nasopharynx, oropharynx, or hypopharynx. Therefore, in the present study, we introduced a small endoscope (endobronchial scope) to perform EUS-FNA sampling from the RLNs via the nasopharynx. However, the sector plane of the bronchoscope probe was too large and long; thus, introducing the probe and manipulating it through the nasal meatus was difficult. Furthermore, puncture and suction led to some mixing of the surrounding tissue or blood into the obtained tissue samples, which could have increased the false-negative rate. Furthermore, because the number of cases was limited, evidence from additional cases should be assessed to verify the reliability of this method.

## Conclusions

In summary, the present pilot study has provided clear evidence that EUS-FNA is a simple, convenient, safe, and effective method to obtain tissue samples from the RLNs for pathological and cytological examination. The technique can be widely used to diagnose or confirm RLN metastasis. In the future, prospective clinical studies with larger cohorts should be planned to further evaluate the safety and efficacy of this method for the diagnosis of suspected RLN metastasis in patients with NPC.

## Additional file

**Additional file 1: Video S1.** Sampling of the retropharyngeal lymph nodes with fine needle aspiration under endoscopic ultrasound guidance in patients with post-radiotherapy nasopharyngeal carcinoma.
